# Reduced serum concentrations of biomarkers reflecting Leydig and Sertoli cell function in male patients with congenital adrenal hyperplasia

**DOI:** 10.1530/EC-23-0073

**Published:** 2023-07-14

**Authors:** Trine Holm Johannsen, Jakob Albrethsen, Vassos Neocleous, Federico Baronio, Martine Cools, Lise Aksglaede, Niels Jørgensen, Peter Christiansen, Meropi Toumba, Pavlos Fanis, Marie Lindhardt Ljubicic, Anders Juul

**Affiliations:** 1 Department of Growth and Reproduction, Copenhagen University Hospital - Rigshospitalet, Copenhagen, Denmark; 2 International Centre for Research and Research Training in Endocrine Disruption of Male Reproduction and Child Health (EDMaRC), Rigshospitalet, University of Copenhagen, Copenhagen, Denmark; 3 The Cyprus Institute of Neurology and Genetics, Department of Molecular Genetics, Function and Therapy, Nicosia, Cyprus; 4 S. Orsola-Malpighi University Hospital, Department of Medical and Surgical Sciences, Bologna, Italy; 5 Department of Pediatrics, Division of Pediatric Endocrinology, Ghent University Hospital and Department of Internal Medicine and Pediatrics, Ghent University, Ghent, Belgium; 6 Pediatric Endocrinology Clinic, Department of Pediatrics, Aretaeio Hospital, Nicosia, Cyprus; 7 Department of Clinical Medicine, University of Copenhagen, Denmark

**Keywords:** LH, INSL3, Testosterone, FSH, inhibin B, AMH

## Abstract

Congenital adrenal hyperplasia (CAH) is a recessive condition that affects the adrenal glands. Despite life-long replacement therapy with glucocorticoids and mineralocorticoids, adult patients with CAH often experience impaired gonadal function. In pubertal boys and in men with CAH, circulating testosterone is produced by the adrenal glands as well as the testicular, steroidogenic cells. In this European two-center study, we evaluated the function of Leydig and Sertoli cells in 61 boys and men with CAH, primarily due to 21-hydroxylase deficiency. Despite conventional hormone replacement therapy, our results indicated a significant reduction in serum concentrations of both Leydig cell-derived hormones (i.e. insulin-like factor 3 (INSL3) and testosterone) and Sertoli cell-derived hormones (i.e. inhibin B and anti-Müllerian hormone) in adult males with CAH. Serum concentrations of INSL3 were particularly reduced in those with testicular adrenal rest tumors. To our knowledge, this is the first study to evaluate circulating INSL3 as a candidate biomarker to monitor Leydig cell function in patients with CAH.

## Introduction

Congenital adrenal hyperplasia (CAH) is a recessive condition that affects the adrenal glands. The condition occurs in 1 out of 14–18,000 births worldwide (1). The most common form of CAH results from mutations in the cytochromep450 family 21 subfamily A member 2 gene (*CYP21A2)*, which leads to 21-hydroxylase deficiency (21-OHD) and may be life-threatening due to a reduced adrenal production of cortisol and aldosterone. The disturbance leads to an increased production of adrenocorticotropic hormone and, thus, an overproduction of adrenal androgens, which may result in virilization, an altered growth pattern, and infertility (2). Treatment of CAH may comprise life-long replacement therapy with glucocorticoids and mineralocorticoids; however, adult patients with CAH often experience inadequate treatment with health sequelae including an impaired gonadal function (3).

One of the most important testicular complications in male patients with CAH is the development of testicular adrenal rest tumors (TARTs) which may be present already in childhood and adolescence (4). The presence of TART constitutes a diagnostic and management challenge. Usually, TARTs are bilaterally localized, benign tumors that have been assumed to derive from aberrant adrenal cells descended together with the testis during fetal life. However, lately, it has been hypothesized that TARTs stem from pluripotent cells as the tumors also have testicular features (5). Associations between high concentrations of adrenocorticotropic hormone and TARTs have been reported, indicating that adrenocorticotropic hormone may promote the development of TARTs, perhaps already in early life (5). However, TARTs have been described independently of disease control, as such other factors may most likely add to their development (6).

The biochemical assessment of gonadal capability in male patients with CAH includes measurements of gonadotropins and testicular and adrenal hormones. The elevated adrenal androgen concentrations may directly suppress luteinizing hormone (LH) and indirectly suppress follicle-stimulating hormone (FSH) by their aromatization to estradiol. The evaluation of Leydig and Sertoli cells has traditionally included measurements of testosterone, inhibin B and anti-Müllerian hormone (AMH). Elevated concentrations of adrenal androgens may result in testosterone concentrations within the reference range despite low LH and FSH concentrations in CAH patients. Thus, additional markers of Leydig cell function that are independent of adrenal androgens are needed in the management of this patient group.

Insulin-like factor 3 (INSL3) is a peptide hormone secreted by the Leydig cells, and it appears to be a new clinical biomarker of Leydig cell function. When compared to testosterone, it has been reported to be more vulnerable to Leydig cell damage (7). Based on an established state-of-the-art liquid chromatography-tandem mass spectrometry (LC-MS/MS) technique for INSL3 quantification with associated male reference ranges (8, 9), we recently showed a dichotomy with lower INSL3 concentrations and higher testosterone concentrations in patients with hypogonadotropic hypogonadism and Klinefelter syndrome (10). Accordingly, it could be speculated that INSL3 is a new candidate in the management of male patients with CAH, and therefore, the present study was commenced on the initiative of the European Reference Network on Rare Endocrine Conditions (https://endo-ern.eu/).

The aim of this study was to assess Leydig and Sertoli cell function in male patients with CAH by evaluating reproductive hormone concentrations, including INSL3, according to sex- and age-related reference ranges and to the presence or absence of TART.

## Material and methods

### Study population

In total, 61 males with CAH (median age: 28.8 years; range: 0.6–55.0 years) were included from two participating tertiary centers, that is, the Department of Growth and Reproduction, Rigshospitalet, Copenhagen, Denmark (*n* = 54) and the Department of Pediatric Endocrinology, Ghent University Hospital, Ghent, Belgium (*n* = 7). The genetically or clinically verified CAH diagnoses comprised 21-OHD (*n* = 59) and 11β-hydroxylase deficiency (*n* = 2).

The following information was obtained from patient records: (i) biochemistry: serum concentrations of LH, FSH, testosterone, 17-hydroxyprogesterone (17-OHP), androstenedione, dehydroepiandrosterone sulfate (DHEAS), inhibin B and AMH; (ii) scrotal ultrasonography: presence of TART up to 6 years before blood sampling (in both participating centers, the protocol for TART screening is on annual basis from puberty onwards); (iii) anthropometrics: height, target height, and weight, and body mass index (BMI) was calculated as weight (kg) divided by height (m) squared. Body surface area (BSA) was calculated according to the formula by the Du Bois method: BSA = 0.007184 × weight (kg)^0.425^ × height (cm)^0.725^ (11); and (iv) hormone replacement therapy: total dose of glucocorticoid in milligrams per BSA per day (patients < 18 years), total dose of glucocorticoid in milligrams per day (patients ≥ 18 years), and total dose of fludrocortisone in micrograms per day (all patients). Doses of glucocorticoid treatment were calculated with the following conversion factors: 20 mg hydrocortisone corresponded to 5 mg prednisolone and 0.75 mg dexamethasone, respectively (12). Thus, the prednisolone effect equaled four times the hydrocortisone effect, and the dexamethasone effect equaled 26.67 times the hydrocortisone effect.

### Hormone assays

All blood samples were stored at −20°C until analysis. As samples were drawn as part of clinical follow-up, the variation in diurnal hormonal secretion was not taken into consideration. In total, 111 samples were included; on average, there were 1.8 (range 1–5) samples per patient. In Danish patients, the samples were drawn as part of clinical follow-up, and except for INSL3, reproductive hormones were analyzed routinely in some of the patients immediately after sampling when ordered by the physician. The numbers of samples without initial request on reproductive hormones included: LH: *n* = 33; androgen group (i.e. testosterone, 17-OHP, androstenedione, and DHEAS): *n* = 2; FSH: *n* = 40; inhibin B: *n* = 41; and AMH: *n* = 98. In all Belgian patients and in Danish samples in whom the total hormone panel had not been requested, the reproductive hormones were measured at the same time. Furthermore, INSL3 was measured in all patient samples at the same time. In total, five reproductive hormone results were not available (FSH: *n* = 1, inhibin B: *n* = 1, AMH: *n* = 3).

Serum concentrations of LH and FSH were determined by two different assays; that is, (i) time-resolved immunofluorometric assays (AutoDelfia; PerkinElmer) as previously described (13), both with limits of detection (LODs) of 0.05 IU/L and inter-assay coefficients of variation (CVs) below 9%; (ii) chemiluminescence immunoassays (Atellica; Siemens Healthineers, Tarrytown, NY, USA) with LODs of LH of 0.07 IU/L and FSH of 0.3 IU/L, respectively, and CVs below 25%. FSH results from the immunofluorometric method were re-calculated to the equivalent results from the chemiluminometric method using an internal correction factor to adjust for instrument bias, while no correction was required between the LH methods. Serum INSL3 was determined by LC-MS/MS as previously reported (8), with a LOD of 0.03 µg/L and a CV below 15%. Serum concentrations of testosterone, 17-OHP, androstenedione, and DHEAS were measured by LC-MS/MS as previously described (14) with limits of quantification (LOQs) of 0.10 nM, 0.19 nM, 0.18 nM, and 48 nM, respectively, and CVs below or equal to 6%. Serum concentrations of inhibin B were measured by an enzyme-linked immunosorbent assay (Beckman Coulter Inhibin B Gen II ELISA, Beckman Coulter, Brea, CA, USA) with a LOD of 3 pg/mL and a CV below 9%. Serum concentrations of AMH were analyzed by a chemiluminescence immunoassay (Access 2, Beckman Coulter) with a LOD of 0.14 nmol/L and a CV below 5%. All analyses were accredited for medical examination from The Danish Accreditation Fund in accordance with the standard DS/EN 15189. Measurements below the lower measurement limits were displayed as LOD/2 or LOQ/2.

### Statistical analyses

To enable comparisons across ages, serum concentrations of reproductive hormones and anthropometric data were standardized to sex- and age-related standard deviation (s.d.) scores using Generalized Additive Model for Location, Scale, and Shape (GAMLSS) statistics that transforms data to follow a parametric distribution: s.d. score = ((*X*/*M*)^*L*^− 1)/(*L* × *S*), where *X* is the measurement, *L* adjusts for skewness (*L* ≠ 0), *M* corresponds to the median, and *S* approximates the CV. Thus, hormone concentrations were standardized to and displayed on previously published reference materials, some of which have been amended according to methodology; that is, LH and FSH (13), INSL3 (9), testosterone, 17-OHP, androstenedione, and DHEAS (15, 16), and inhibin B and AMH (17). Due to missing reference data in specific age ranges, standardization of hormone concentrations was not possible in all patients < 18 years (Table 1). Biochemical s.d. scores with values below −7 or above 7 were recoded to −7 and 7, respectively. Anthropometric data were standardized to height s.d. scores, target height s.d. scores, height corrected for target height s.d. scores (height s.d. scores minus target height s.d. scores), and BMI s.d. scores. In Danish patients, standardization was performed according to Danish reference data (18), and in Belgian patients, standardization was performed according to Belgian reference data (19). Standardization of anthropometric data was not possible in all patients < 18 years (due to missing reference data in specific age ranges) and in all patients ≥ 18 years (due to lack of measurements) (Table 1).

**Table 1:** Baseline characteristics in male patients with congenital adrenal hyperplasia (CAH). Except for testicular adrenal rest tumor (TART), data are shown as medians and interquartile ranges. *n* refers to the number of available data within each variable.

	Male CAH patients < 18 years	*P*-value < 18 years	Male CAH patients ≥ 18 years	*P*-value ≥ 18 years
*N*	18	-	43	-
Age (years)	7.8 (4.1 to 12.3), *n* = 18	-	35.1 (26.2 to 46.3), *n* = 43	-
LH (SDS)	0.13 (−1.03 to 0.46), *n* = 13	0.753	−0.32 (−1.42 to 0.83), *n* = 43	0.214
INSL3 (SDS)	0.79 (−0.68 to 0.88), *n* = 15	0.191	−1.38 (−2.07 to −1.03), *n* = 43	**<0.001**
Testosterone (SDS)	−0.43 (−1.03 to 0.09), *n* = 10	0.241	−1.21 (−2.26 to −0.15), *n* = 43	**<0.001**
FSH (SDS)	−0.20 (−1.64 to 0.64), *n* = 13	0.552	0.13 (−0.87 to 1.01), *n* = 43	0.856
Inhibin B (SDS)	−0.37 (−1.09 to 0.37), *n* = 18	0.064	−1.07 (−1.84 to −0.59), *n* = 43	**<0.001**
AMH (SDS)	−0.52 (−1.05 to 0.18), *n* = 18	**0.031**	−0.97 (−2.17 to −0.29), *n* = 43	**<0.001**
17-OHP (SDS)	4.68 (2.81 to 6.60), *n* = 15	**0.001**	6.39 (1.20 to 7.00), *n* = 43	**<0.001**
Androstenedione (SDS)	1.60 (0.30 to 2.44), *n* = 15	**0.017**	1.42 (−1.90 to 3.89), *n* = 43	**0.022**
DHEAS (SDS)	−1.31 (−1.85 to 0.52), *n* = 15	0.281	−3.17 (−3.79 to −1.55), *n* = 43	**<0.001**
TART^a^	1, *n* = 8	-	19, *n* = 38	-
Height (SDS)	0.41 (−0.41 to 0.90), *n* = 18	0.257	−1.42 (−1.98 to −0.68), *n* = 42	**<0.001**
H-SDS minus TH-SDS (SDS)	0.54 (−1.04 to 1.12), *n* = 18	0.528	−0.84 (−1.46 to −0.25), *n* = 24	**<0.001**
Body mass index (SDS)	0.91 (−0.27 to 1.77), *n* = 16	0.059	1.55 (0.68 to 2.16), *n* = 42	**<0.001**
Hydrocortisone, HC (mg/day)	-	-	27.8 (24.4 to 32.7), *n* = 42	-
HC pr. BSA (mg/m^2^/day)	11.1 (8.0 to 12.5), *n* = 17	-	-	-
Fludrocortisone (µg/day)	90.0 (70.0 to 110), *n* = 13	-	200 (100 to 200), *n* = 33	-

^a^Number of patients with TART having a testicular sonography performed within the last 6 years; HC dose was calculated from glucocorticoid doses with conversion factors (20 mg HC = 5 mg prednisolone; 20 mg HC = 0.75 mg dexamethasone).

17-OHP, 17-hydroxyprogesterone; AMH, anti-Müllerian hormone; BSA, body surface area; DHEAS, dehydroepiandrosterone sulfate; FSH, follicle-stimulating hormone; H-SDS minus TH-SDS, height SDS minus target height SDS; INSL3, insulin-like factor 3; IQR, interquartile range; LH, luteinizing hormone; SDS, standard deviation score.

Medians were calculated for biochemical, anthropometric, and treatment markers to hinder skewing of data by single patients contributing with more samples. The one-sample Wilcoxon signed-rank test was used when comparing patient s.d. scores to zero (reference material), and the Mann–Whitney *U* test was used when comparing s.d. scores in patients with and without TART. Data were reported as medians and interquartile ranges (IQRs). s.d. scores ranging from −2 to +2 were considered normal, and *P*-values below 0.05 were considered statistically significant. Figures and statistical analyses were performed using Excel (Microsoft Office 365), GraphPad Prism (version 9), and IBM Statistics SPSS (version 25).

## Results

Baseline characteristics of the CAH patients are shown in Table 1.

### Reproductive hormones related to Leydig cell function

**Figure 1:**
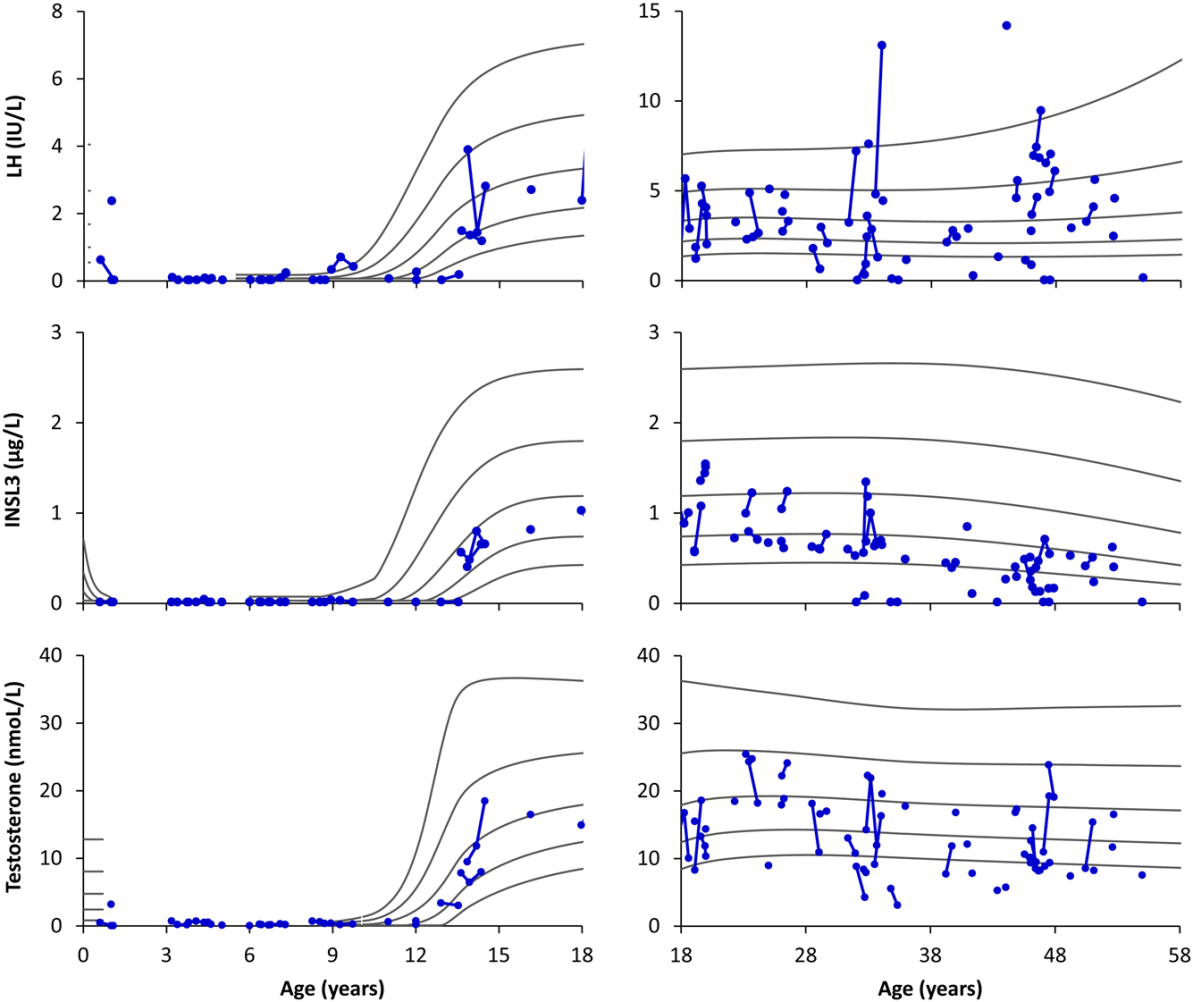
Serum concentrations of luteinizing hormone (LH, upper panels), insulin-like factor 3 (INSL3, middle panels) and testosterone (lower panels) according to age in 61 male patients with congenital adrenal hyperplasia (blue dots). Each blue dot represents one patient with consecutive results connected by blue lines. Black lines indicate −2 standard deviation (s.d.), −1 s.d., 0 s.d., +1 s.d., and +2 s.d., respectively.

In patients < 18 years, the absolute serum concentrations of Leydig cell markers were within the reference ranges in most cases (Fig. 1). The corresponding percentages of patients with median s.d. scores within the normal range of ±2 s.d. were LH = 77%, INSL3 = 93%, and testosterone = 80%, and no statistically significant differences were observed when comparing s.d. scores to the reference (Table 1). In patients ≥ 18 years, the absolute serum concentrations of LH were distributed across the whole reference range, while INSL3 and testosterone concentrations were below and in the lower part of the reference range (Fig. 1). The corresponding percentages of patients with median s.d. scores within the normal range were LH = 72%, INSL3 = 74%, and testosterone = 70%, and median INSL3 and testosterone s.d. scores were significantly lower when compared to the reference (Table 1).

### Reproductive hormones related to Sertoli cell function

**Figure 2:**
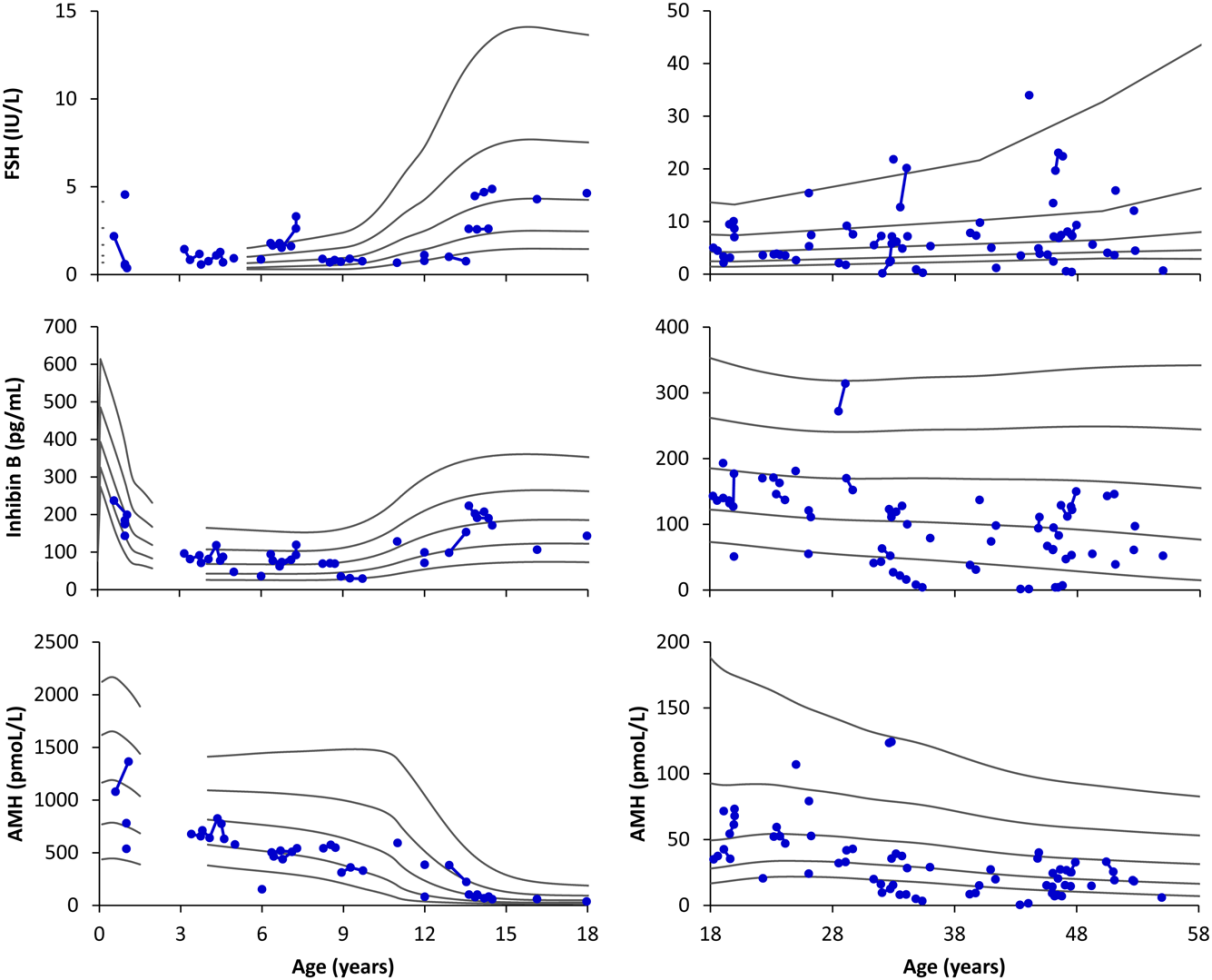
Serum concentrations of follicle-stimulating hormone (FSH, upper panels), inhibin B (middle panels), and anti-Müllerian hormone (AMH, lower panels) according to age in 61 male patients with congenital adrenal hyperplasia (blue dots). Each blue dot represents one patient with consecutive results connected by blue lines. Black lines indicate −2 standard deviation (s.d.), −1 s.d., 0 s.d., +1 s.d., and +2 s.d., respectively.

In patients < 18 years, the absolute serum concentrations of Sertoli cell markers were within the reference ranges in most cases (Fig. 2), although AMH concentrations tended to be in the lower part of the range. The corresponding percentages of patients with median s.d. scores within the normal range were FSH = 69%, inhibin B = 100%, and AMH = 94%, and AMH s.d. scores were significantly lower when compared to the reference (Table 1). In patients ≥ 18 years, the absolute serum concentrations of FSH were distributed across the whole reference range, while inhibin B and AMH concentrations were below and in the lower part of the reference (Fig. 2). The corresponding percentages of patients with median s.d. scores within the normal range were FSH = 79%, inhibin B = 77%, and AMH = 72%, and median inhibin B and AMH s.d. scores were significantly lower when compared to the reference (Table 1).

### Adrenal hormones

Irrespective of age, median s.d. scores of 17-OHP and androstenedione were significantly higher than the reference, and in the patients ≥ 18 years, the median s.d. scores of DHEAS were significantly lower than the reference (Table 1). Thus, only 7% of patients < 18 years and 23% of patients ≥ 18 years had median s.d. scores within the normal range of 17-OHP. The corresponding percentages were 67 and 28% for androstenedione, and 60 and 26% for DHEAS. As it is impossible to discriminate between testosterone from adrenal vs testicular origin, testosterone s.d. scores are only reported in the section earlier.

### Testicular adrenal rest tumor

**Figure 3:**
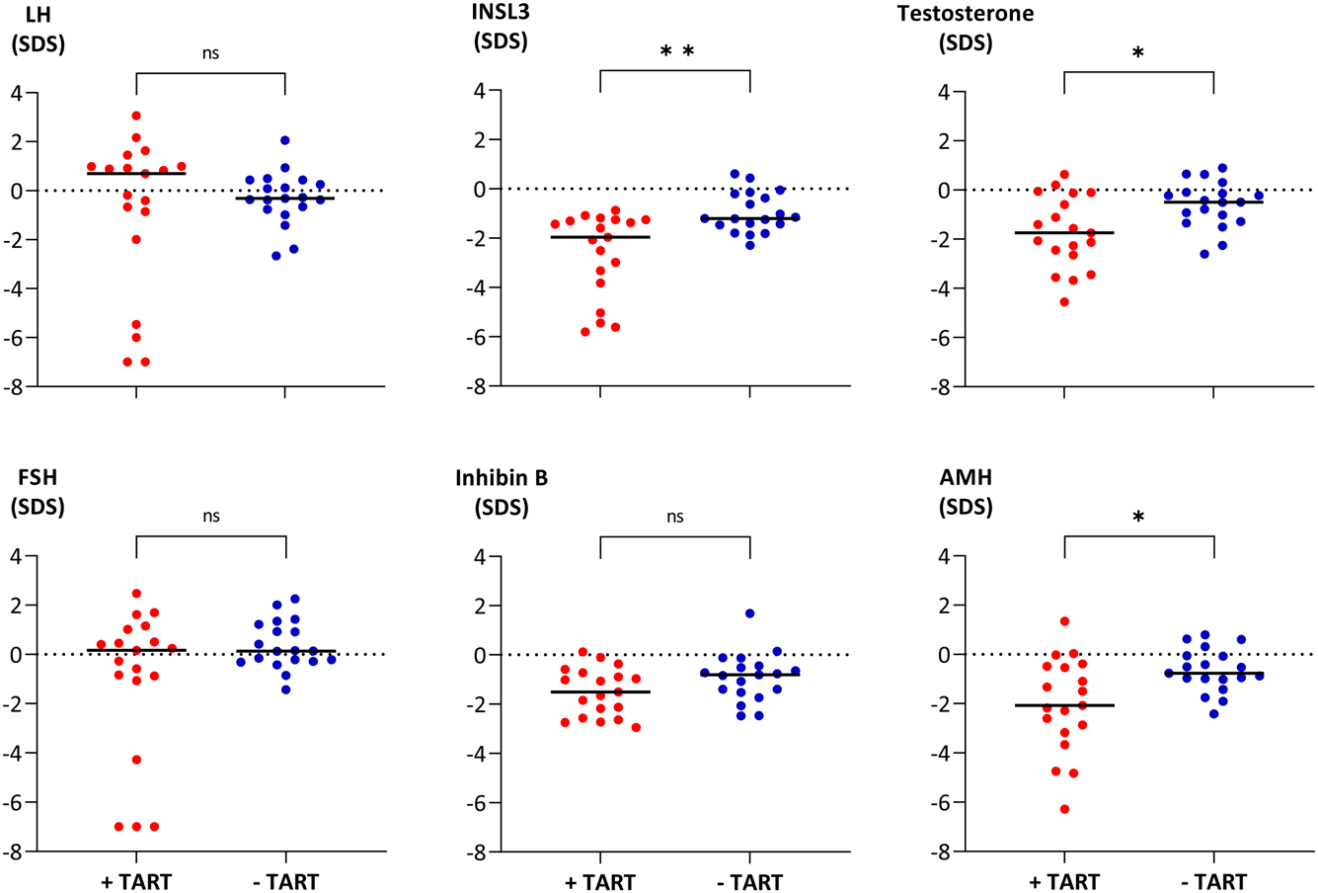
Serum concentrations of luteinizing hormone (LH), insulin-like factor (INSL3), testosterone, follicle-stimulating hormone (FSH), inhibin B, and anti-Müllerian hormone (AMH) expressed as median S.D. scores (SDSs) in patients with congenital adrenal hyperplasia aged ≥ 18 years. Patients are classified according to the presence (red dots, *n* = 19) or absence (blue dots, *n* = 19) of testicular adrenal rest tumors (TARTs) up to 6 years before blood sampling, with each dot representing one patient. Black, marked lines indicate medians of the groups, and dotted lines indicate 0 SDS. ns, non-significant, *P-value < 0.05, **P-value < 0.01.

**Figure 4:**
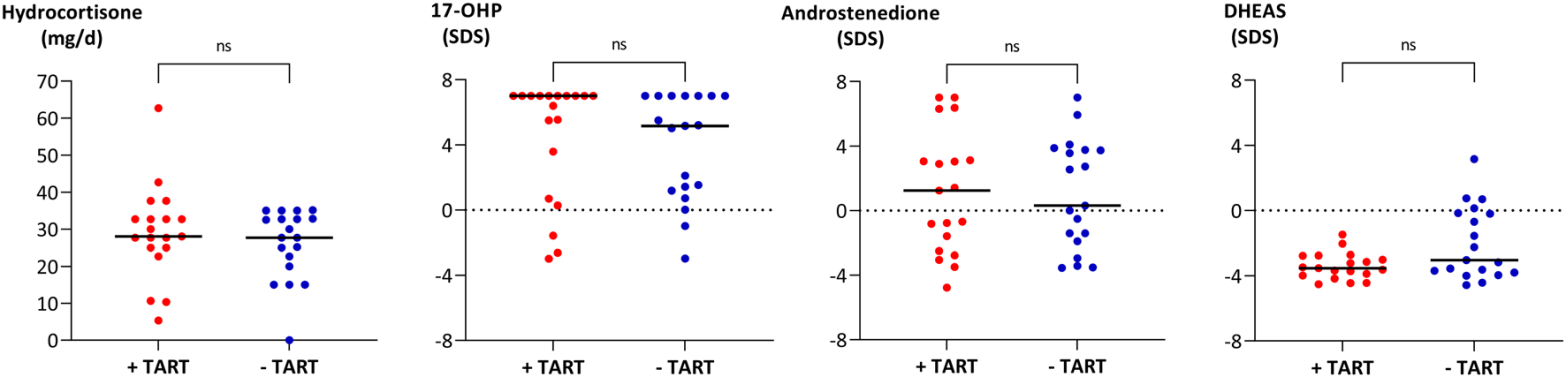
Total dose of hydrocortisone shown as milligrams per day and serum concentrations of 17-hydroxyprogesterone (17-OHP), androstenedione, and dehydroepiandrosterone sulfate (DHEAS) expressed as median S.D. scores (SDSs) in patients with congenital adrenal hyperplasia aged ≥ 18 years. Patients are classified according to the presence (red dots, *n* = 19) or absence (blue dots, *n* = 19) of testicular adrenal rest tumors (TARTs) up to 6 years before blood sampling, with each dot representing one patient. Black, marked lines indicate medians of the groups, and dotted lines indicate 0 SDS. ns = non-significant.

**Figure 5:**
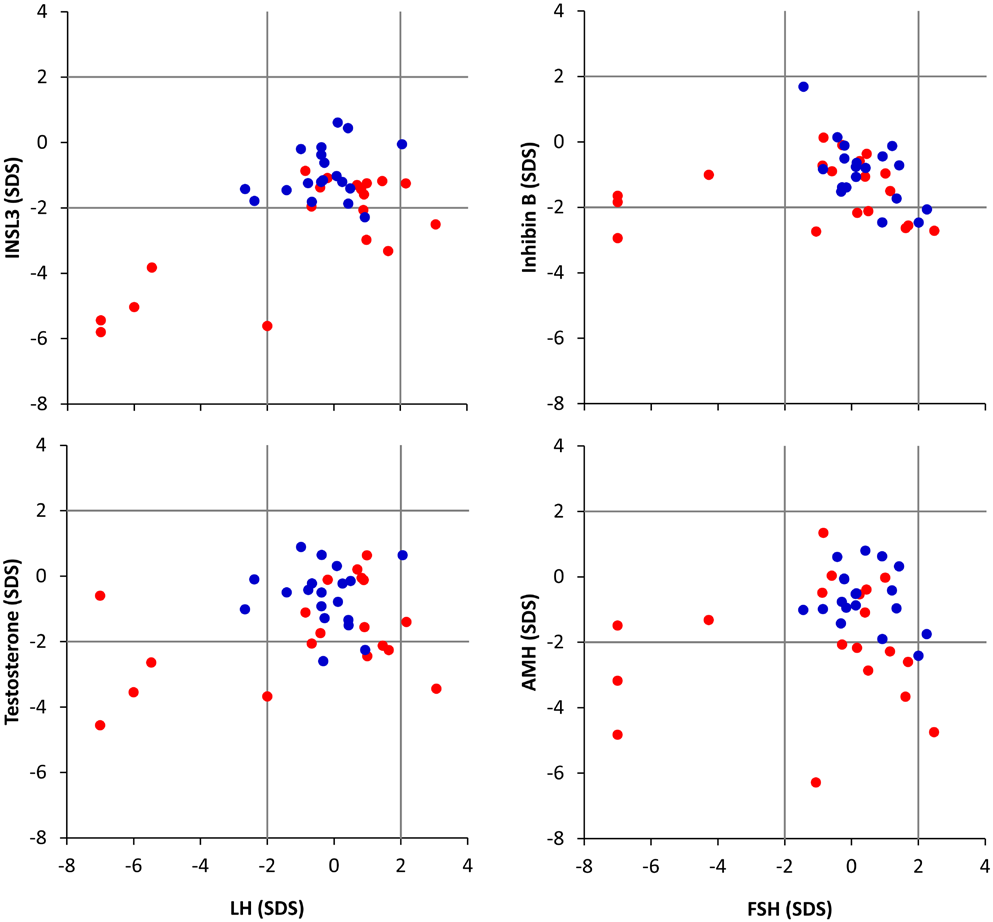
Serum concentrations of reproductive hormones expressed as median S.D. scores (SDSs) in patients with congenital adrenal hyperplasia aged ≥ 18 years. Left panel: serum concentrations of insulin-like factor 3 (INSL3) and testosterone as a function of concentrations of luteinizing hormone (LH), respectively. Right panel: serum concentrations of inhibin B and anti-Müllerian hormone (AMH) as a function of follicle-stimulating hormone (FSH), respectively. Patients are classified according to the presence (red dots, *n* = 19) or absence (blue dots, *n* = 19) of testicular adrenal rest tumors (TARTs) up to 6 years before blood sampling, with each dot representing one patient. Gray lines indicate ±2 SDS.

In the patient group < 18 years, TART was reported in one patient (aged 13 years), corresponding to 12.5% of reported examinations with testicular sonography in this group. In patients ≥ 18 years, the equivalent fraction of TART was 50% (Table 1). When comparing patients ≥ 18 years with TART to patients without TART, no significant differences in median s.d. scores were observed in serum concentrations of LH, FSH, inhibin B, 17-OHP, androstenedione and DHEAS, respectively, nor in the daily hydrocortisone-equivalent dose (Figs. 3 and 4). Despite overlaps between the groups, patients with TART had lower median s.d. scores of both INSL3 (*P* = 0.002), testosterone (*P* = 0.019), and AMH (*P* = 0.016) concentrations as compared to patients without TART. In spite of the low s.d. scores of INSL3, testosterone, and AMH, the large majority of patients ≥ 18 years had median LH and FSH s.d. scores within ±2 s.d. (Fig. 5). The very low median LH and FSH s.d. scores originated from the same four patients with TART.

### Anthropometrics

In patients < 18 years, the median s.d. scores of height, height minus target height, and BMI were within the normal range in 94, 100, and 81%, respectively, and no statistically significant differences were observed when comparing median s.d. scores to the reference (Table 1). The corresponding percentages of patients ≥ 18 years with median s.d. scores within the normal range were height = 76%, height minus target height = 88%, and BMI = 64%; median height and height minus target height s.d. scores were significantly lower when compared to the reference, and median BMI s.d. scores were significantly higher when compared to the reference.

### Hormone replacement therapy

Among patients < 18 years, 17 (94%) were treated with hydrocortisone and 13 (72%) with fludrocortisone (Table 1). Overall, the median doses of hydrocortisone and fludrocortisone were in accordance with the clinical practice guideline from the Endocrine Society for maintenance treatment in growing patients with CAH (i.e. hydrocortisone: 10–15 mg/m^2^ per day; fludrocortisone: 50–200 µg per day (20)). Among patients ≥ 18 years, 42 (98%) were treated with hydrocortisone or its equivalents (hydrocortisone: *n* = 14; hydrocortisone + dexamethasone: *n* = 23; dexamethasone: *n* = 2; and prednisolone: *n* = 3) and 33 (77%) with fludrocortisone. Overall, the combined median doses of hydrocortisone were higher than recommended by the guideline for maintenance treatment in fully grown patients with CAH (i.e. 15–25 mg per day). This was also observed in patients treated with hydrocortisone only (median daily dose = 25 (19–35) mg). The median doses of fludrocortisone were in the upper part of the recommended dose interval (i.e. 50–200 µg per day).

## Discussion

In this study, from two European centers, we evaluated the Leydig and Sertoli cell function in 61 boys and men with CAH, primarily due to 21-OHD. Using accredited and highly sensitive analytical methods, the concentrations of gonadotropins, Leydig cell hormones, Sertoli cell hormones, and adrenal hormones were measured. All concentrations were evaluated both as absolute values and as relative values by means of age-dependent s.d. scores according to Danish sex- and age-specific reference ranges and relating to the presence of TART. Although the majority of CAH patients in the study had reproductive hormone concentrations within age-specific reference ranges, a substantial minority of the adult patients had significantly reduced concentrations of both Leydig and Sertoli cell hormones. To our knowledge, this is the first report on concentrations of circulating INSL3 in CAH patients.

In terms of Leydig cell capacity, the picture of low testosterone concentrations in adult males with CAH has previously been reported (21). Yet, the serum concentration of testosterone may also be within or above the normal range, and since the testosterone concentration reflects a combination of both testicular and adrenal derivation, and the concentration of LH to some degree may indicate whether an overproduction of adrenal-derived testosterone is present, the evaluation of Leydig cell function in male patients with CAH is challenging. INSL3 is exclusively and constitutively produced in the Leydig cells, so the circulating concentration of this hormone depends on the number and differentiation of the Leydig cells (22); thus, INSL3 may add to the evaluation of Leydig cell function. Previous studies support INSL3 as a more sensitive and direct marker of Leydig cell capacity, as compared to testosterone. For example, as opposed to testosterone, INSL3 may not fully recover after a relatively long period of testicular suppression (7, 23). In line with this, in the present study, INSL3 was significantly decreased in the adult population, which may suggest it as a possible diagnostic tool for Leydig cell damage in adult male patients with CAH. It is previously reported that INSL3 secretion depends on long-term stimulation by LH (24), and it could also be speculated that the low INSL3 concentrations simply were the result of suppressed LH concentrations. However, as shown in Fig. 5, more than half of the patients with INSL3 below 2 s.d. in fact had LH concentrations within or above the reference range. In contrast, the patients < 18 years did not differ from the reference with respect to the Leydig cell markers.

In terms of Sertoli cell capacity, low inhibin B concentrations have also previously been reported in some adult males with CAH (21). In this study, 40 of the 43 adult patients had median s.d. scores of inhibin B below the average for age (corresponding to a s.d. of 0). As serum concentrations of inhibin B correlate with spermatogenesis in healthy men (25, 26), our findings may suggest that spermatogenesis is affected in the adult patients in this study. In line with this observation, 36 of the 43 adult patients had median s.d. scores of AMH below 0 s.d. Similarly, 12 of 18 patients < 18 years had median scores of AMH below 0; thus, AMH may be considered an additive diagnostic tool in the evaluation of Sertoli cell capacity in boys with CAH. In contrast, inhibin B s.d. scores did not differ statistically from the reference material. It should be emphasized that no firm conclusion can be drawn on this matter without confirmation of our findings by a more reliable method to assess spermatogenesis such as semen analysis.

Evaluation of 17-OHP, androstenedione, and DHEAS were reported independently in this study even though these markers also resemble the Leydig cell production. Serum concentrations of 17-OHP and androstenedione were significantly increased both in patients < 18 years and in patients ≥ 18 years. According to the Endocrine Society guideline, the concentrations of these hormones should be in the upper range or slightly elevated in sufficiently treated patients (20). On the contrary, serum concentrations of DHEAS were significantly decreased, which previously has been reported in this patient group (27). Thus, as the height may be regarded as a proxy for disease control in childhood (28), and as the patients < 18 years had height s.d. scores not differing from the reference, they may be regarded as being reasonably well titrated on their replacement therapy. In contrast, as a group the adult population had significantly decreased height s.d. scores, which may indicate insufficient disease control or excess glucocorticoid exposure during childhood and/or adolescence. Moreover, in the adult patients, weak, significant correlations were observed between median s.d. scores of height and LH and INSL3, respectively (data not shown); that is, a decreased height correlated to low hormone concentrations and vice versa. However, it is unknown whether the decreased LH and INSL3 concentrations reflect long-term negative effects from inadequate disease control in childhood that affected growth, or whether they rather are due to insufficient disease control in adulthood and the correlation thus is a coincidental find.

TARTs are known complications of CAH, and among those who underwent testicular sonography in this study, TART was described in half of the patients ≥ 18 years and in one (aged 13 years) of eight patients < 18 years. Our findings are in line with a recent study, in which the prevalence of TART verified by ultrasound or magnetic resonance imaging during the last 10 years was summarized; an average prevalence of TART of 46% in male adults and of 25% in adolescents was reported (5). TART is primarily described in inadequately treated patients (29), but they have also been observed in satisfactorily treated patients, suggesting other contributing factors to tumor development (6). In line with this, we found no statistically significant differences in s.d. scores of gonadotropins, 17-OHP, androstenedione, or DHEAS, nor in the daily dose of glucocorticoid, between adult patients with and without the presence of TART. Although both groups had low s.d. scores of INSL3, testosterone, and AMH, the lowest concentrations were observed in the TART group. In accordance with this, our previous immunohistochemical study revealed that INSL3 was expressed in normal human Leydig cells and Leydig cell tumors, but not in TART (30). Thus, to exclude TART, testicular ultrasonography may be considered in patients with INSL3 or AMH concentrations below −2 s.d.

The strengths of this study included: (i) detailed clinical and biochemical data in 61 patients with CAH, primarily due to 21-OHD; (ii) all samples were measured in the same laboratory with highly sensitive state-of the-art-methodology; and (iii) the application of s.d. scores that allowed for comparisons of hormone concentrations and anthropometrics across ages. However, this study also had limitations: (i) the retrospective design of the study resulted in some missing data and (ii) the samples were drawn as part of clinical follow-up without consideration of the diurnal variation of hormone secretion.

In conclusion, we observed a significant reduction in Leydig and Sertoli cell hormones in adult males with CAH, most of whom received conventional hormone replacement therapy with glucocorticoids and mineralocorticoids. Furthermore, serum concentrations of INSL3, testosterone, and AMH were reduced in patients with TART compared to patients without, indicating a more pronounced impairment in testicular function in this group. We, therefore, propose that the Leydig cell-specific hormone INSL3 merits further investigation as a candidate biomarker to monitor Leydig cell capacity in male patients with CAH.

## Declaration of interest

There is no conflict of interest that could be perceived as prejudicing the impartiality of the research reported.

## Funding

The Frimodt-Heinike Foundation (F-23653), and The Torben and Alice Frimodt Foundation (F-23023-02).

## Ethical approvals

Access to and management of Danish patient data was approved by the Danish Patient Safety Authority (no. 3-3013-1376/1/), the Team for Medical Records Research, Centre for Health, the Capital Region of Denmark (R-22031906), and the Danish Data Protection Agency (no. 2015-235 (I-Suite no. 04204) and P-2022-364). In Denmark, the permission for hormone measurements was approved as part of a quality assurance project at Copenhagen University Hospital – Rigshospitalet (no. 20032415). In Belgium, the study was approved by the institutional ethical committee (ID: 2008/098).
